# Generating Implementation Research Knowledge for Scaling Lifestyle Modification Intervention to Control Hypertension and Diabetes: Lessons from the CoCo-HD Study in Kerala

**DOI:** 10.5334/gh.1585

**Published:** 2026-09-29

**Authors:** Nabil Abdul Majeed, Sandra Sebastian, Henna Kondeth, Kripa Selvarajan Valsa Shiby, Akshay Parambilan Kandi, Renu Vijayakumaran Nair Prasanna, Sonu Soman Ammariparambil, Bipin Gopal, Abey Sushan, Muraleedharan Sarojini Manu, Kavumpurathu Raman Thankappan, Panniyammakal Jeemon

**Affiliations:** 1 Sree Chitra Tirunal Institute for Medical Sciences and Technology (SCTIMST), Thiruvananthapuram, India; 2 International Institute for Population Sciences (IIPS), Mumbai, India; 3 Airport Health Organisation (APHO), Trivandrum, India; 4 Centre for Chronic Disease Control (CCDC), New Delhi, India; 5 Department of Health & Family Welfare, Government of Kerala, Trivandrum, India; 6 P K Das Institute of Medical Sciences, Palakkad, India; 7 Amrita Vishwa Vidyapeetham, Amrita Institute of Medical Sciences, Kochi, India

**Keywords:** Knowledge generation, implementation research, structured lifestyle modification, hypertension, diabetes, peer-led intervention, Kerala, knowledge transformation

## Abstract

This article describes how a structured lifestyle modification intervention for hypertension and diabetes was scaled within Kerala’s health system, focusing on how implementation knowledge was generated and embedded in routine practice. Grounded in an implementation research approach, the work documents how evidence-informed strategies were continuously adapted through stakeholder engagement, peer-led delivery, and supportive institutional processes. Drawing on qualitative data from stakeholder consultations, document review, and field observations in *Janakeeya Arogya Kendrams* (basic unit of primary health care centres in Kerala), the article shows how local context, social learning, and power dynamics shaped iterative learning cycles. The findings outline key facilitators and barriers to knowledge use in practice. It offers practical insights for strengthening sustainable non-communicable disease prevention and control programmes in similar settings.

## Background

Non-communicable diseases (NCDs) like hypertension and diabetes drive premature illness and death worldwide (1). In Kerala, India, NCDs drive substantial premature morbidity, straining public health services (2). The state has recently reinforced community-based primary care through Janakeeya Arogya Kendrams (JAKs), which serve as the primary platform for the National Programme for Prevention and Control of Non-Communicable Diseases (NP-NCD) at the community level (Online Supplement, Box 1 & Figure 1) (3). Yet, control rates for both hypertension and diabetes remain low (4). The Community Control of Hypertension and Diabetes (CoCo-HD) project expands peer-supported structured lifestyle modification programmes to foster lasting behavioural changes and improve control rates in Kerala (5). Drawing on field experiences, this paper describes knowledge generation as a cyclical, collaborative process involving government, health workers, communities, and researchers to adapt lifestyle evidence into practical, culturally relevant strategies for better NCD control in Kerala. This manuscript specifically aims to document the qualitative process of knowledge co-creation (6), examining how existing evidence on lifestyle modification was interpreted and reshaped through interactions among stakeholders.

### Conceptual Framework

Drawing on social constructivist principles (7), our study views knowledge as co-created through interactions among researchers, health workers, policymakers, and communities, rather than as fixed information transferred one-way from experts. This process brings together two key forms of knowledge: attained knowledge, encompassing scientific evidence and formal programme guidelines, and embodied knowledge, reflecting lived experiences, community beliefs, and local practices (Figure 1). This intersection yields adapted knowledge that remains scientifically sound, while being contextually realistic. We used ‘transformation’ rather than ‘translation’ (8,9) to emphasise that the process was not a linear or one-directional conversion of a fixed body of evidence into practice. It demonstrates a bidirectional and iterative process in which already-validated clinical evidence was repeatedly interpreted, adapted, negotiated, and reshaped as it moved across different levels and contexts of the health system. In this paper, we put together our contribution as an empirical account of how established clinical knowledge acquires institutional legitimacy and is progressively made locally relevant and actionable (Online Supplement Box 2).

**Figure 1:**
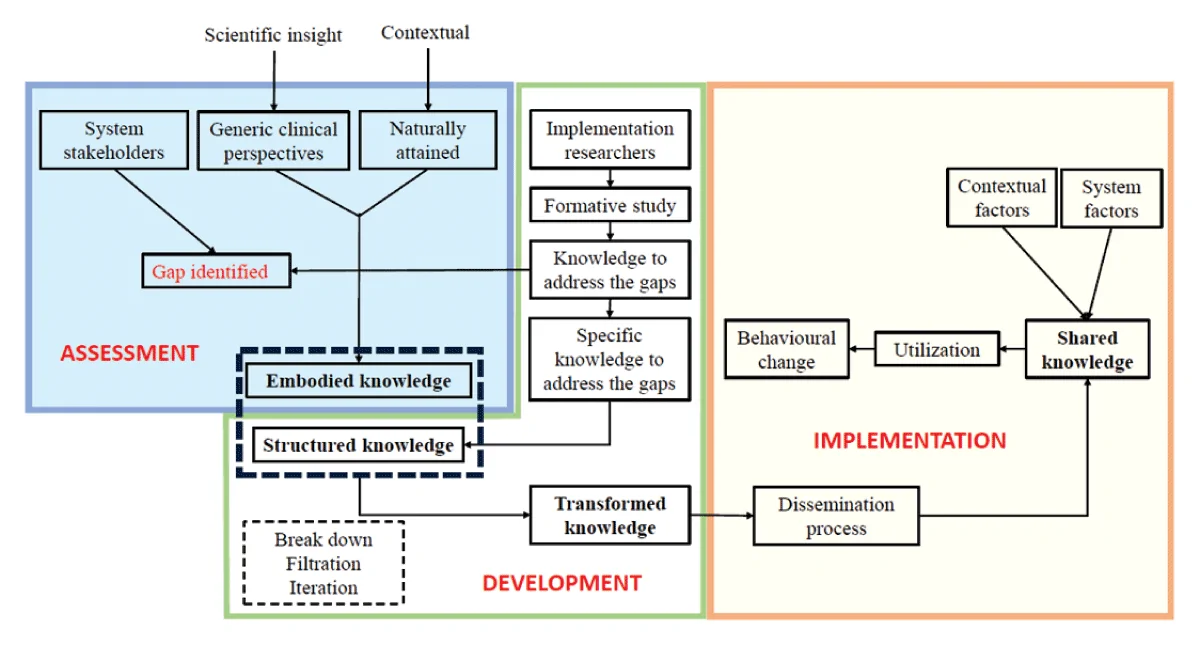
Conceptual framework: knowledge transformation process.

Our conceptual framework clearly distinguishes two types of elements in the structured lifestyle modification intervention. One includes consistent features across settings, such as positive peer influence, organised group sessions, monitoring frequency, and group composition, while the other involves locally shaped understandings and responses influenced by social, cultural, demographic, and organisational factors that affect how lifestyle-related knowledge is interpreted and applied. Guided by three principles from implementation research: acceptability, or how well the intervention aligns with community values; adoptability, or the health system’s readiness and capacity to implement it; and appropriateness, or the extent to which the strategies are accessible, understandable, and usable for the target population (10), the article highlights how knowledge is continually refashioned through contextual influences, social learning, and differences in power.

## Methods

Our study employed a qualitative implementation research approach, incorporating multiple methods to capture how knowledge is created, communicated, and adapted within the CoCo-HD initiative. Stakeholder consultations were conducted with policymakers, state and district health administrators, mid-level service providers (MLSPs), JAK team members, and community representatives to gather insights into how knowledge is generated and transferred across different levels of the system. A total of 52 participants were interviewed/consulted in the process, including state-level officials (*n* = 6), district-level officials (*n* = 8), medical officers (*n* = 7), primary care providers (*n* = 15), healthcare activists and other experts (*n* = 4), and beneficiaries (*n* = 1). Data collection took place over a period of five months, from May to September, 2023, in four districts of the state, along with the state headquarters, and was conducted by trained members of the study team. The transcripts were independently reviewed and coded by two researchers to ensure consistency before framework analysis. Field observations were carried out during pilot peer-led group meetings implementing the SLM intervention, enabling the research team to document how information was conveyed, how participants engaged with it, and how peer interactions shaped understanding. A systematic review of programme documents, including training manuals, information, education and communication (IEC) materials, and organisational guidelines under the NP-NCD programme was undertaken to map how knowledge is produced, interpreted, and modified within the health system. Member checking (11) and peer debriefing (12) were held with frontline workers and programme staff to validate emerging interpretations, ensure accuracy, and enable iterative refinement of findings (detailed methodology is given in the Online Supplement, Box 3).

The data were analysed using the principles of framework analysis (13). The framework covered thematic aspects of contextual influences (such as health system organisation, workforce responsibilities, and community participation), stages of knowledge transformation, socio-political dynamics, and systemic barriers (Online Supplement, Table 1).

## Results

### Collaborative and iterative knowledge generation

Knowledge generation for SLM intervention was a collaborative, iterative process that drew insights from multi-level stakeholders. State, district, and local officials supplied policy direction, institutional support, and resources, aligning the intervention with public health priorities. MLSPs and JAK teams provided practical insights from community interactions, identifying cultural norms, challenges, and opportunities for lifestyle acceptance. Community members, including people living with hypertension and diabetes, contributed their lived experiences and perceptions, which shaped how lifestyle messages were understood and acted upon. Researchers synthesised this knowledge into a framework that blends evidence with local realities. This multidirectional dialogue refined components through feedback, fostering relevance, ownership, and adaptation rather than top-down prescription (Online Supplement, Table 1, refer codes SEKJ; SEKC).

### Adapting scientific evidence into practical, localised knowledge

Scientific recommendations on diet, physical activity, and medication adherence were first reviewed and adapted to determine their suitability for local contexts, with regional food habits, cultural norms, and daily routines taken into account (Online Supplement, Examples of Knowledge Transformation Process). Insights from frontline health workers were then incorporated to fine-tune intervention components, for instance, adjusting peer group composition to reflect local gender dynamics and social relationships. This was followed by the development of culturally resonant communication materials, including participant handbooks, memorable, straightforward slogans, and adaptable facilitation approaches that enabled peer leaders to guide discussions effectively. The adapted knowledge was reinforced through regular monthly peer-led sessions conducted by trained MLSPs at JAKs, promoting interactive learning and shared problem-solving rather than one-way instruction. Through repeated engagement and collective discussion, these groups gradually grew into self-sustaining spaces where participants not only absorbed new information but also motivated one another, acting as both learners and drivers of behaviour change (Online Supplement, Table 1, refer to codes GITK, KDCA, RSAISU, & GCSHI).

### Institutionalisation of knowledge within the health system

Strong institutional backing proved essential for the effective implementation and continuity of the SLM intervention. The Kerala government formally incorporated the intervention into the NP-NCD programme structure, a step that helped bypass administrative delays and created a pathway for scaling the initiative across districts. Training platforms were established to prepare MLSPs and JAK teams not just as implementers but as active facilitators of knowledge, strengthening their capacity to guide peer groups and support community engagement. In addition, monitoring and evaluation systems were built into routine programme operations, generating a steady flow of feedback that allowed for continuous refinement of intervention strategies. This pattern of embedding can be understood through three complementary theoretical lenses. Institutional isomorphism (14) explains the organisational form that this integration took, while the political economy of knowledge production (7) helps explain whose endorsement and authority were necessary to confer legitimacy on the knowledge. From an implementation research perspective, Normalisation Process Theory (15) further explains the everyday processes and mechanisms through which this knowledge became embedded within routine practice (Online Supplement, Box 4 & Table 1, refer to codes HSS, IKHP, SLPS & PEKP).

### Enablers and barriers to knowledge translation

The study revealed several factors that supported effective knowledge translation and highlighted important lessons for strengthening community-based interventions. Kerala’s high literacy levels and strong community networks created favourable conditions for understanding health messages and sustaining peer support, demonstrating that leveraging existing social capital is a critical enabler. Government commitment ensured steady resources and smooth programme integration, showing that institutional ownership is essential for long-term continuity. The engagement and preparedness of MLSPs and JAK teams reinforced their role as key knowledge facilitators, underscoring the need to invest in frontline worker capacity. At the same time, several barriers emerged, offering valuable insights. Irregular participation in peer groups, driven by work demands, migration, or stigma, indicated that interventions must be flexible and sensitive to livelihood realities. Deep-rooted dietary patterns and lifestyle behaviours proved challenging to change, underscoring the need for behavioural interventions to account for cultural norms and household routines. Logistical constraints faced by frontline workers, including time limitations and material shortages, underscore that successful implementation depends on strengthening supportive systems and addressing operational bottlenecks (Online Supplement, Table 1, refer to codes CESC, MLSP & BKTU).

## Conclusion

Our findings demonstrate that knowledge generation for lifestyle interventions is not a simple transfer of information from experts to communities, but a socially constructed, continuously evolving process shaped by interactions among multiple stakeholders. Peer-led models played a central role in this process by creating spaces where individuals could share experiences, relate scientific guidance to their daily lives, and collectively work through challenges. This blending of formal knowledge with the lived realities of communities proved essential for fostering meaningful and sustained behaviour change. Embedding the intervention within Kerala’s established health system further strengthened institutional memory, governance structures, and accountability mechanisms, all of which are crucial for expanding and maintaining such programmes on a scale. The study also illustrates how institutional priorities and existing power relations influence what is recognised as legitimate evidence in public health decision-making, underscoring the broader political economy that shapes knowledge production.

Overall, the generation and application of knowledge to support structured lifestyle modification for hypertension and diabetes control in Kerala emerged as a dynamic, participatory effort involving government leadership, frontline health worker engagement, community participation, and research synthesis. This collaborative approach ensured that scientific recommendations were not only technically sound but also locally meaningful, feasible to implement, and supported by the health system. Sustained scaling of such interventions will require continued investment in frontline capacities, strengthening peer-support networks, and fostering flexible and inclusive processes that allow knowledge to be adapted as contexts evolve (Online Supplement Box 5). Future work should examine the long-term effects of this approach and its potential to inform similar initiatives in other settings (Online Supplement Box 6).

## Additional File

The additional file for this article can be found as follows:

10.5334/gh.1585.s1 Supplementary File. Supplementary tables, figures, and boxes providing additional methodological details and supporting results for the study.
