# Supplementary material for: Generating Implementation Research Knowledge for Scaling Lifestyle Modification Intervention to Control Hypertension and Diabetes: Lessons from the CoCo-HD Study in Kerala
Source: Glob Heart. 2026 Sep 29;21(1):70. doi: 10.5334/gh.1585 (PMC13629543; doi:10.5334/gh.1585)
Supplement: Supplementary file 1 [file gh-21-1-1585-s1.pdf]

Online Supplement

Title

Generating Implementation Research Knowledge for Scaling Lifestyle Modification Intervention to Control Hypertension and Diabetes: Lessons from the CoCo-HD study in Kerala

Nabil Abdul Majeed[1], Sandra Sebastian[2], Henna Kondeth[1], Kripa Selvarajan Valsa Shiby[3], Akshay Parambilan Kandi[4], Renu Vijayakumaran Nair Prasanna[5], Sonu Soman Ammariparambil[6], Bipin Gopal[5], Abey Sushan[5], Muraleedharan Sarojini Manu[5], Kavumpurathu Raman Thankappan[7] and Panniyammakal Jeemon[1].

1. Sree Chitra Tirunal Institute for Medical Sciences and Technology (SCTIMST), Thiruvananthapuram, India
2. International Institute for Population Sciences (IIPS), Mumbai, India
3. Airport Health Organisation (APHO), Trivandrum, India
4. Centre for Chronic Disease Control (CCDC), New Delhi, India
5. Department of Health & Family Welfare, Government of Kerala, Trivandrum, India
6. P K Das Institute of Medical Sciences, Palakkad, India
7. Amrita Vishwa Vidyapeetham, Amrita Institute of Medical Sciences, Kochi, India

Corresponding Author

Panniyammakal Jeemon

Additional Professor

Sree Chitra Tirunal Institute for Medical Sciences and Technology (SCTIMST), Thiruvananthapuram, India

Email Address: jeemon@sctimst.ac.in

Contact Number: +91-9899246116

Box (1) Health system support for the scale-up of structured lifestyle modification intervention delivery through the Janakeeya Arogya Kendrams – JAKs in Kerala[1]

JAKs or Janakeeya Aroya Kendrams, otherwise known operationally as sub-centres or Health and Wellness Centres, represent the foundational community-level unit within Kerala's tiered public health system. Each JAK serves a population of 3000 - 5000 beneficiaries. Each JAK unit is staffed with Mid-Level Service Providers (MLSPs) who are mostly trained nurses, along with Junior Public Health Nurses and Junior Health Inspectors. They serve as grassroot level institution for essential primary NCD care, bridging clinical services with community outreach. Within the Structured Lifestyle Modification Intervention (SLMI) program, JAKs serve as the operational epicentre for community-based peer group interventions targeting control of hypertension and diabetes among beneficiaries. MLSPs trained in a phased manner across the state, form and lead peer groups of 15 plus participants per JAK, conducting 12 structured sessions annually on lifestyle modification on topics like diet, exercise, self-monitoring, medication adherence and related topics. The JAK team facilitate the peer group intervention by identifying participants, securing venues, liaising with local self-bodies and ensuring that the sessions occur monthly across all the JAKs. The program is monitored and supervised by supervisory staff at different levels who report to the Medical Officers, Block-level officials and District administrative officials. Digitally generated and updated data is reported to the State headquarters monthly.[2]

Fig (1) Organisation of the health system for management of non-communicable diseases at the state, district and block level in Kerala[1]

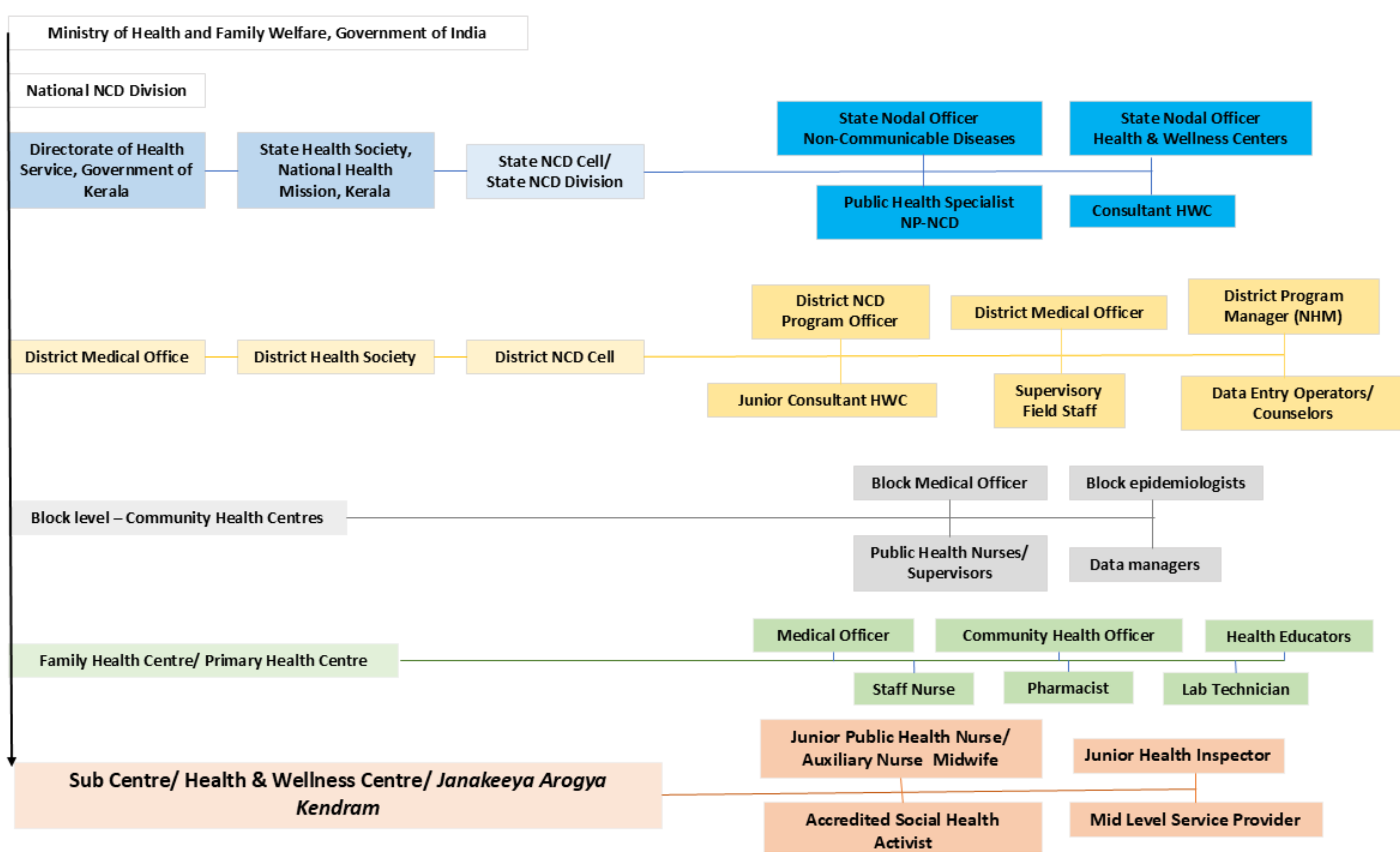

Box (2) Theoretical Rationale: Why "Knowledge Transformation" Rather Than "Knowledge Translation"

We use the term knowledge transformation, rather than the more established knowledge translation, to describe the process documented in this paper, and set out our reasoning here in full, since we recognise this diverges from the dominant vocabulary of implementation science.

Most knowledge translation (KT) frameworks, for example, the Knowledge-to-Action cycle, or diffusion-of-innovations models conceptualise an existing, largely fixed body of evidence that must be moved, with minimal alteration to its substance, from a producer (researchers, guideline bodies) to a user (clinicians, communities). Uptake is understood chiefly as a problem of dissemination, format and channel: the content of the knowledge is treated as already settled, and the work of "translation" lies principally in packaging and delivering it effectively to a new audience.[3,4]

Our data did not fit this model well, and what stakeholder groups ultimately delivered to beneficiaries was not simply the pre-existing clinical evidence base on lifestyle modification. Instead, the content itself changed in form and substance as it moved through the system. MLSPs and JAK team members embedded contextual detail; medication-adherence routines suited to local daily rhythms, culturally specific quantification of dietary intake rather than formal calorie counts, and locally resonant slogans and repetition strategies that altered what counted as usable, actionable knowledge for beneficiaries. This is consistent with Mode 2 knowledge production,[5] in which knowledge is generated through application-oriented, transdisciplinary engagement between researchers, health-system actors and communities, rather than through a one-directional expert-to-user pipeline;[6] and with social constructivist accounts of knowledge, in which legitimacy is achieved only once knowledge is situated within specific social, cultural and institutional settings.

We therefore treat translation as a special, largely content-conservative case of a broader transformation process and reserve the term transformation for processes such as the one documented here in which content, form and legitimacy are jointly reshaped through iterative interaction between attained knowledge (formal, scientific, system-level) and embodied knowledge (experiential, contextual, community-level). This distinction underpins the paper's contribution by moving beyond demonstrating the efficacy of structured lifestyle modification, which has already been substantially established by prior trials such as PROLIFIC[7] and Kerala Diabetes Prevention Programme[8] to provide an empirical account of the mechanisms captured by our framework, namely acceptability, adoptability, and appropriateness, through which established evidence gains local legitimacy, becomes usable in routine practice, and can ultimately inform adaptation and implementation in other settings.

Box (3) Extended Methods: Sampling, Data Collection, and Quality Assurance

Study design

This study used a qualitative implementation research design informed by the principles of framework analysis,[9] nested within the broader Community Control of Hypertension and Diabetes (CoCo-HD) type 3 hybrid effectiveness–implementation trial.[10] The qualitative component reported in the manuscript was designed specifically to document how implementation knowledge for the structured lifestyle modification (SLM) component was generated, adapted, and institutionalised, complementing the quantitative outcome evaluation reported under the parent trial protocol.

Sampling strategy

A purposive, maximum-variation sampling strategy was used to capture perspectives across all levels of the health system engaged in delivering the SLM intervention, from policy formulation through to frontline delivery and lived experience. Participants were selected to reflect variation in institutional role, geographic location within Kerala, and (for beneficiaries) gender, duration of participation in peer groups, and level of engagement with the intervention. The final sample of 52 participants comprised state-level officials (n = 6) responsible for NP-NCD programme stewardship and policy direction; district-level officials (n = 8) responsible for operationalising the SLM component across Janakeeya Arogya Kendrams (JAKs); medical officers (n = 7) supervising clinical integration of the intervention; primary care providers (n = 15), including Mid-Level Service Providers (MLSPs), Junior Public Health Nurses (JPHNs), Junior Health Inspectors (JHIs) and Accredited Social Health Activists (ASHAs) directly delivering peer-led sessions; healthcare activists and other experts (n = 4) providing cross-cutting technical input; and beneficiaries (n = 12) enrolled in SLM peer groups.

Data collection instruments and period

Semi-structured interview guides and a structured field-observation checklist were developed based on the study's conceptual framework (attained versus embodied knowledge; the acceptability, adoptability and appropriateness domains) and piloted with a small number of respondents before wider use. This ensured comparability of core domains probed across stakeholder categories while allowing flexibility to explore issues specific to each group's role in the knowledge-generation process. The relevant fieldwork for the study was done over a period of five months during the year 2023. Interviews were conducted in Malayalam and/or English according to participant preference, audio-recorded with informed consent, and transcribed verbatim. Interviews conducted in Malayalam were translated into English by bilingual members of the study team; a sample of translated transcripts was independently checked against the original audio to verify semantic equivalence.

Data collectors and training

Interviews and field observations were conducted by trained members of the study's qualitative research team, working under the supervision of the co-investigators. A common training protocol, covering interview technique, probing strategies, and research ethics, was used to standardise data collection across the team and across stakeholder categories.

Quality assurance and trustworthiness

Consistent with established criteria for rigour in qualitative research, several strategies were used to strengthen the trustworthiness of the findings.

Credibility was enhanced through methodological triangulation across stakeholder interviews, field observations of peer-group sessions, and systematic review of programme documents. Member checking was conducted with frontline implementers, and peer debriefing was undertaken with health-system stakeholders representing multiple cadres to assess the interpretation and consistency of the findings.

Dependability was strengthened through the use of a shared coding framework derived from the conceptual model. Two researchers independently double-coded a subset of transcripts, and any discrepancies were resolved through discussion and consensus. Regular research team meetings were also conducted to review the analytic process and maintain a documented audit trail of coding decisions, interpretations, and subsequent modifications.

Confirmability was addressed through reflexive documentation maintained by the field team. These notes explicitly considered the research team's dual role as researchers involved in the intervention and as technical team members and trainers, as well as the potential influence of these roles on data collection, participant responses, and interpretation of the findings.

Transferability was supported by providing a detailed description of the institutional and contextual setting, presented in Box 1 and Figure 1. This contextual detail enables readers to assess the relevance and potential applicability of the findings to comparable health-system settings.

Data analysis

Data were analysed using framework analysis,[9] an approach well suited to applied, multi-stakeholder implementation research. Analysis proceeded through five stages. First, the research team familiarised themselves with the full body of data; 52 stakeholder transcripts, field observation notes from peer-group sessions, and programme documents (training manuals and IEC materials), reading and re-reading material to gain an overall sense of the dataset before formal coding began. Second, a coding framework was developed jointly, informed by the study's conceptual model of attained and embodied knowledge and by the acceptability, adoptability and appropriateness domains guiding the intervention; it yielded thirteen codes organised under three overarching categories: Contextual Factors, Knowledge Transformation Process, and Epistemological and Theoretical Considerations (Table 1). Third, transcripts were indexed against this coding framework, with a subset independently double-coded by two researchers and discrepancies resolved through discussion to ensure consistency of application across the dataset.

Fourth, coded data were charted into a framework matrix (Table 1), cross-tabulating each code against stakeholder group and against the four overarching results themes of collaborative and iterative knowledge generation; adapting scientific evidence into practical, localised knowledge; institutionalisation of knowledge within the health system; and enablers and barriers to knowledge translation, allowing systematic comparison of how each theme was expressed differently across state officials, district officials, medical officers, primary care providers, healthcare activists and beneficiaries. Fifth, the research team mapped and interpreted the charted data to identify recurring patterns, points of divergence between

stakeholder groups (for example, contrasting institutional and frontline perspectives on the SLPS and BKTU codes), and explanatory linkages across levels of the health system, iteratively revisiting the conceptual framework to interpret how generic scientific evidence was progressively transformed into locally legitimised, actionable knowledge. Consistent with established criteria for rigour in qualitative research,[11] this process was supported throughout by peer debriefing[12], member checking[13] with frontline implementers, and a documented audit trail of analytic decisions.

Box (4) Institutionalisation of Knowledge: Theoretical Elaboration using three complementary lenses

A central finding of this study is that knowledge did not become durable, scalable, or practically usable solely because it was scientifically robust or effectively communicated. Rather, its sustained use and potential for wider adoption depended on its recognition and endorsement within the institutions in which it was intended to operate. We elaborate here, in greater theoretical depth than was possible within the main text, on the concept of knowledge institutionalisation as it relates to our findings

Institutional Isomorphism

Institutional theory holds that organisations, and the knowledge that flows through them, gain legitimacy not purely from technical merit, but through conformity with, and endorsement by, recognised institutional structures a dynamic sometimes described as institutional isomorphism.[14] In our data, this was visible in the Kerala state government's decision to formally integrate the SLM component into the existing NP-NCD programme structure. This act of integration did several things simultaneously: it converted a project-specific intervention into a recognised, budgeted part of routine government health delivery; it created a pathway for scaling that bypassed the administrative friction typically associated with piloting novel, externally funded interventions; and it signalled to frontline health workers (MLSPs and JAK teams) that the SLM component carried the same institutional authority as other, longer-established NP-NCD activities.

This institutionalisation was also path-dependent. The intervention was not delivered through a parallel or purpose-built structure, but through the pre-existing JAK platform and the newly created MLSP cadre. This meant that the form the "transformed knowledge" ultimately took how it was packaged into training modules, participant handbooks, and peer-group formats was constrained and shaped by the capacities, workflows and professional identities already present within the health system. Institutionalisation was therefore not a neutral, downstream event that occurred after knowledge was generated; it actively shaped what forms of knowledge could be generated and legitimised in the first place.

Political economy of knowledge production

We also note a political-economy dimension to this process, consistent with critical accounts of knowledge production in public health[3]; what counts as legitimate, "ready-to-scale" knowledge is not determined solely by evidentiary strength, but by whether it aligns with institutional priorities and in this case, the state's interest in strengthening NCD control without a substantial additional resource burden and whether it can be absorbed into existing organisational routines. This helps explain why some elements of the intervention (for example, structured peer-group delivery, which aligned with existing outreach mechanisms) were rapidly institutionalised, while others requiring a larger departure from existing practice (for example, the specific training MLSPs required to deliver "structured" content that went beyond common-sense advice) needed more deliberate, sustained work to gain endorsement.

Normalisation Process Theory

This pattern is also well captured by Normalization Process Theory (NPT), a sociological theory developed specifically to explain how new practices become routinely embedded in everyday work.[15] NPT identifies four interacting mechanisms through which this occurs: coherence, the sense-making work by which a new practice is framed as meaningful and distinct from existing work; cognitive participation, the enrolment and

buy-in of the key actors who must carry the practice forward; collective action, the operational work of actually, enacting the practice within existing workflows; and reflexive monitoring, the ongoing appraisal and refinement of the practice once in use. Read through this lens, the SLM component's institutionalisation reflects successful coherence-building (framing SLM as an extension of, rather than an addition to, existing NP-NCD work) and cognitive participation (enrolling MLSPs and JAK teams as knowledge facilitators rather than passive implementers), while the monitoring and evaluation systems described in the main text function as the reflexive-monitoring mechanism that has sustained the practice over time. NPT therefore offers another precise theoretical vocabulary for describing the specific work observed in the study data.

Box (5) Positioning the Study's Contribution and Addressing Generalisability

The contribution of this paper is not the intervention itself, but the demonstration of how a real (not experimental) health system converts already-credible scientific knowledge into a form that is simultaneously scientifically defensible and locally actionable, at population scale. This is a different question from “does structured lifestyle modification work” which is already substantially answered by prior trials, including PROLIFIC[7] and the Kerala Diabetes Prevention Programme[8] and was closer to “how does a health system generate the specific, contextualised and institutionally legitimised form of that knowledge needed to deliver it at population scale.”

This framing also allows to address a genuine tension between the need for generalisable, standardised knowledge (to justify scale-up, funding and replication) and the reality, borne out in our data, that local consensus, terrain, workforce configuration and cultural norms shaped what worked. The approach tried to resolve this tension analytically, not by claiming the intervention is either fully standardised or fully local, but by distinguishing, as our conceptual framework does, between the properties of the intervention that remained stable across contexts (positive peer influence, structured communication with a defined timeframe, regular monitoring, inclusivity, adaptive reusability of the structure) and the components that were context-specific (peer-group composition, session frequency, workforce configuration). It is the properties, not the specific components, that we argue are generalisable and transferable to other settings; the components must be re-derived locally through a comparable process of stakeholder engagement.

Box (6) Transferable Lessons for Similar Settings

Building on the enablers and barriers identified, we set out the following as more explicit, transferable lessons for programmes seeking to scale community-based non-communicable disease interventions in comparable low and middle income settings.

1. Treat knowledge generation as an explicit, resourced activity, rather than an assumed by-product of implementation. Dedicated time and personnel for stakeholder consultation, document review, and iterative material development were necessary conditions for the SLM component to reach a form that health workers and beneficiaries could use.
2. Invest early in securing institutional (government) ownership. Programmes that remain formally external to existing government structures are more vulnerable to discontinuity; A formal integration into an existing national programme, as occurred in our study with the NP-NCD programme, created durable administrative and financial backing.
3. Build frontline workers' capacity as knowledge facilitators, not only as implementers. Training MLSPs and JAK teams to understand and adapt the rationale behind SLM content, rather than simply deliver a fixed script, was central to the knowledge remaining responsive to local conditions over time.
4. Build monitoring and evaluation into routine programme operations from the outset, rather than treating it as a research add-on. It both generates the feedback needed for continuous refinement and helps align state and implementer expectations about what data collection is for.
5. Anchor communication materials in locally resonant language, repetition, and everyday routines rather than technical accuracy alone. Structured slogans, handbook recap segments, and concrete behavioural guidance (for example, medication timing tied to daily routines rather than abstract dosage instructions) were more consistently taken up than generic health messaging.
6. Expect, and plan for, a period of institutional negotiation. Aligning a new intervention with existing bureaucratic structures, workforce cadres, and reporting requirements takes deliberate relationship-building with state and district officials.

Table (1): Coding Framework for Implementation Research and Knowledge Generation

Theme	Sub-theme	Coding Category	Code / Title	Description	Illustrative Quotations
Collaborative and Iterative Knowledge Generation	Multi-level stakeholder input in knowledge generation	Knowledge Transformation Process	SEKG — Stakeholder Engagement in Knowledge Generation	Explores the role of health workers, policymakers, and researchers in shaping intervention strategies.	*"The JAK team is actually very informed; they are the ones who are capable of providing knowledge at the root level. Once they are trained, they should be able to reach people with the right information."* (District Administrative Officer-3)<br>*"We, as researchers, are trying to understand what information needs to be converted, and what the possible ways are to practically put together transformed information."* (Research Team Member-1)<br>*"Everyone on the team should have the same set of information handy. This will enable uniformity. It is important to bring everyone's ideas and doubts together." (*District Administrative Officer- 2)
	Multidirectional dialogue, co-production and feedback	Epistemological & Theoretical Considerations	SCKC — Social Constructivism & Knowledge Co-Production	Investigates how knowledge is collaboratively developed and shared.	*"There is a need to understand the perspectives of multiple stakeholders, including beneficiaries, on lifestyle modification in general. For building on needful information, bring their own perceived knowledge and understand that first."* (Health care activist, Jeevathalam)<br>*"Everyone has generalised embodied knowledge. It can be scientific or self-perceived. This is what we need to convert to transformed information on lifestyle modification."* (Research Team Member-2)

Theme	Sub-theme	Coding Category	Code / Title	Description	Illustrative Quotations
					"No one should have the upper hand in knowledge; all have multiple information. Health people have their own knowledge. Common people have their own knowledge. They have their doubts also. Both need to be considered." (Local Governing Body Member - 3)
Adapting Scientific Evidence into Practical, Localised Knowledge	Converting generic scientific evidence into transferable, actionable knowledge	Knowledge Transformation Process	GITK — Generic Information to Transferable Knowledge	Investigates how scientific data is contextualised and adapted for community-level interventions.	*"We already have detailed lifestyle guidelines, but they must be converted into simple, stepwise messages that our field teams can use with the community month after month."* (State Level Official-1)<br>*"It is not that people don't know things. It is about understanding what is right. For that, we should be able to bring their (people's) own perceived knowledge, understand that first…what they know is correct, how much misinformation they have, and then give them the IEC. Then only it works. That's what I have felt."* (Senior public health supervisor)
	Cultural and contextual adaptation of communication materials	Knowledge Transformation Process	KDCA — Knowledge Dissemination & Cultural Adaptation	Studies the translation of intervention knowledge into culturally appropriate formats.	*"We have information on diet, the plate method, calorie calculations, and so on. Those people who can do that— it's fine. But laypeople need information they understand. The fruits we suggest, as per the guidelines, may not be available locally. We should say what can be done with what is available."* (Medical Officer-4)

Theme	Sub-theme	Coding Category	Code / Title	Description	Illustrative Quotations
					"We can inform them which foods contain carbohydrates and to cut down on the portion they eat as of now, and replace them with vegetables or fruits. But not everyone will be able to calculate their calorie [intake] and eat accordingly. So we need a way to say that, acceptable to laypeople." (Research team member-2)
	Tailoring communication to community language and expectations	Epistemological & Theoretical Considerations	RSAISU — Realist Study Approach in Intervention Scale-Up	Examines the underlying mechanisms that drive intervention success.	*"People have their own language, and those who communicate with them in their language, which they can understand easily, are what they expect."* (District Administrative Officer-3)<br>*"We should tell them (beneficiaries) what THEY want to listen to, how to put scientific knowledge into that, which is what guides program success."* (State Level Administrative Officer-3)
	Generalisability and replicability of adapted materials beyond Kerala	Epistemological & Theoretical Considerations	GCSHI — Generalizability vs. Context-Specificity in Health Interventions	Assesses the scalability of the intervention beyond Kerala.	*"The kind of materials generated should be made in such a way that they can be used in multiple contexts, even beyond our state. The modules should be used not only for SLM but for any intervention on diabetes or hypertension. This should be a replicable model."* (State level Administrative Official-1)
Institutionalisation of Knowledge	Health system structure and	Contextual Factors	HSS — Health System Structure	Examines the existing healthcare	*"Yes, it is possible to integrate the program into the JAK model. We have a new cadre, MLSPs. One of their major foci is NCD. So,*

Theme	Sub-theme	Coding Category	Code / Title	Description	Illustrative Quotations
within the Health System	integration with JAKs			delivery models, their integration with JAKs, and their role in NCD prevention.	*we can incorporate it. They need training."* (State level Official-1)<br>*"We now have NCD clinics every day at our institute. We pass information, but the time limit is an issue. OPDs are very busy. We need easy-to-communicate templates."* (Medical Officer-2)
	Formal embedding of SLM within the NP-NCD programme	Knowledge Transformation Process	IKHP — Institutionalisation of Knowledge within Health Programs	Examines how SLM interventions are embedded into Kerala's NP-NCD program.	*"Integrate with JAKs, which is the only way to reach the maximum number of people. And whatever way you try otherwise will fail later. And it should come under the program structure of the NCD program and go along with that to be successful."* (State Administrative Officer-1)<br>*"In JAK, we have mandates to form patient groups and give topic-specific sessions to the beneficiaries. The SLM program should be part of the patient group."* (State Administrative Officer-2)
	State-level policy, funding and institutional backing	Contextual Factors	SLPS — State-Level Policy and Support	Analyses government policies, funding mechanisms, and institutional backing for the SLM intervention.	*"We may need to convince the stakeholders, but the important thing is, we need to give more emphasis on control rates (of hypertension and diabetes)…starting so many dialysis centres is not something we should be feeling good about. We should not let them (patients) reach there. So, we need this kind of intervention."* (State level Official-1)<br>*"We can integrate such a programme with our existing patient group program of Health*

Theme	Sub-theme	Coding Category	Code / Title	Description	Illustrative Quotations
					and Wellness Centres. What we need is good modules and easy-to-communicate information…[we] have provisions to use funds under definitive heads of NCD training and FC funds." (State level Official-2)<br><br>*"We know so many things, but we need ways to say things. For that, it should come through the system. Then only will it be accepted by our people (health workers). We know to treat NCDs, we have medicines, but lifestyle modification is so low. It is because we don't know how to tell. It is important to have modules within the program, funds to train and clear-cut training schedules. Then information will be uniform, and only then will there be a consolidated mechanism."* (Medical Officer-3)
	Political economy and power dynamics shaping institutional buy-in	Epistemological & Theoretical Considerations	PEKP — Political Economy of Knowledge Production	Explores the influence of power dynamics in shaping health interventions.	*"We need to convince the stakeholders who are conducting the program and the government initially. Then only this works. They should understand how this SLM initiative is different and capable of giving better information to people. If that doesn't work well, the process is difficult to take off."* (State level stakeholder-2)<br><br>*"Our supervisory public health staff and medical officers — they should find it appropriate. If they don't find this good enough to communicate, we should consider that something is lacking in the information*

Theme	Sub-theme	Coding Category	Code / Title	Description	Illustrative Quotations
					generated. So their opinion must be included." (District level official-4)
Enablers and Barriers to Knowledge Translation	Community engagement and social capital (enabler)	Contextual Factors	CESC — Community Engagement and Social Capital	Assesses the involvement of local communities in implementing and sustaining interventions.	*"People know things quite well. They read a lot. They have information from so many sources, good and bad. They ask about everything. What I am saying is that people are receptive to information."* (Health care activist, Jeevathalam)<br><br>*"LSD members are trusted in our areas — if they say this event is important for us, people consider it. It may not be right among all Ward members. Some are very influential, and they actively participate. If they are part of the program, people would engage easily."* (Health care Provider-5)<br><br>*"People are sharp. When health people say things (knowledge on diseases), they consider them as real information. New, new information should come from health people."* (LSGD member-1)
	Frontline workforce capacity — MLSPs (enabler)	Contextual Factors	MLSP — Workforce Composition & Role of Mid-Level Service Providers (MLSPs)	Evaluates the contribution of MLSPs in knowledge dissemination and patient engagement.	*"…there is always a possibility of communicating with the people. Our ASHAs are very efficient. JHI and the entire JAK team know everyone in their field area. Now we have MLSPs, and they can be assigned new responsibilities. They are trained for community-based programs, like patient groups. So, we have a good cadre structure in our system."* (District level Official-1)

Theme	Sub-theme	Coding Category	Code / Title	Description	Illustrative Quotations
					"We were given training. But when we go to the field, they (beneficiaries) ask a lot of questions. There is always something new coming up in the field. Simple and effective information will be very helpful for us. People need it to be simple." (Health Care Provider-2)
	Barriers to participation and uptake (barrier)	Contextual Factors	BKTU — Barriers to Knowledge Transfer and Uptake	Identifies factors that hinder knowledge dissemination and patient adherence.	*"Our patients won't come to the centre, especially if we keep it (peer group meetings) during morning hours. They won't compromise work and come. We need to keep information interesting so that they come. Or else it will be the same people coming for all programs."* (Health care provider-3)<br><br>*"Males are reluctant to come. It will be all females. Many would be working, and they would make excuses. They don't want others to know they have BP (hypertension)."* (Health care provider-2)<br><br>*"...making them come to the meeting is difficult. Some would ask straight away what I will get if I come to the meetings. Are there any incentives? Lifestyle modification is needed, but the way we communicate should be convincing."* (Health care provider-4)

Examples of Knowledge Transformation Process

Example 1: Knowledge Transformation Process on Medication Adherence

Example 2: Knowledge Transformation Process on Exercise and Healthy Diet

Example 3: Knowledge Transformation Process on Smoking and Alcohol Cessation

EXAMPLE 1: MEDICATION ADHERENCE

"Despite multiple methods employed, medication adherence among beneficiaries remains an issue"

System Stakeholder perspectives & responses

- *Regular supply of medicines*
- *Daily NCD clinics at Primary Health Centres*
- *Awareness creation through public health staff and frontline health workers*

- *Limited interaction time with patients*
- *Lack of aids to facilitate easy communication*
- *Suboptimal clinical handover during consultation.*

GENERIC CLINICAL PERSPECTIVES

- Adherence to Guideline-Directed Medical Therapy is critical for achieving optimal control of blood pressure and glycemic levels
- Poor adherence is one of the leading causes of uncontrolled hypertension and diabetes, resulting in increased risk of cardiovascular events, kidney disease, stroke, and mortality
- Consistent medication use improves overall treatment outcomes and reduces long-term healthcare costs

Embodied knowledge

Facts

- Even after prescribing multiple medicines, the blood pressure remains uncontrolled
- Once medication is started for hypertension, it is lifelong
- Avoiding sugar eliminates the need for diabetes medication to a large extent

NATURALLY ATTAINED

Myths

- Skipping medications for a day or two will not affect the health
- Multiple medications are unnecessary or excessive
- Lifelong medication is not required
- Treatment could be stopped when one feel better
- When blood sugar and BP remain normal for a few months, it is safe to discontinue the medicines or decrease doses by oneself
- Lifestyle diseases are conditions that should be managed only through diet and exercise, questioning the need for medication

EXAMPLE 1: MEDICATION ADHERENCE

Extracts from Transformed Knowledge

Knowledge

Importance and advantages of taking medications in the right dose at the right time

- Medicines help keep blood pressure and blood sugar within a safe range, preventing silent damage to organs
- Consistent or regular medication use reduces the risk of heart attacks, strokes, kidney failure, nerve damage, and vision loss
- When taken regularly, medicines make one feel more energetic, reduce tiredness, and improve overall well-being
- Proper control of BP and sugar prevents long-term problems or complications, allowing one to perform normal daily activities and work, and to maintain quality of life
- Medicines work best along with lifestyle changes like exercise, diet control, and quitting smoking/alcohol

Why Multiple Medicines Are Needed

- Several organs control BP and sugar, so different medicines are needed to manage them effectively
- Using multiple medicines does not mean the disease is severe; it simply means the treatment is targeting different parts of the body together to keep you safe

Why You Shouldn't Skip Medicines

- BP and sugar often rise quietly, without any signs, so you may feel normal even when levels are dangerously high
- Missing even a few doses can lead to sudden spikes or an increase in BP or blood sugar
- Repeatedly skipping diabetes medicines can cause blood sugar to rise so much that insulin injections may become necessary
- Uncontrolled BP increases the risk of stroke, heart attack, kidney damage, and bleeding in the brain
- Uncontrolled diabetes can damage the eyes, kidneys, nerves, heart, and delay wound healing
- Minor discomforts or mild side effects are far less risky than the serious problems or complications that occur when medicines are skipped

Action

Essential tips to remember about medications

- Know your medications' names, uses, brand names, and side effects
- Always keep a list of your medications
- Ensure you have an adequate supply of your drugs, and don't wait until you're completely out to refill your medicines
- Take your medicines at the prescribed dose and time every day
- Keep a medication calendar to help you stay organised
- Note the dosage you take each time
- If you forget to take a dose, take it as soon as you remember
- Do not reduce the dosage to save money. Take all medications in the prescribed dosage to get the full benefit
- Do not take any medication or herbal treatment without your doctor's approval
- Consider using a pillbox to organise your medications. Mark each day of the week for easy identification, and fill it out at the beginning of each week
- Keep your medications and prescriptions easily accessible when travelling
- Keep extra medication for emergency use during travel.
- Set alarms on your mobile phones as reminders for taking medications
- Seek the help of family and friends to remind you of taking medications on time

EXAMPLE 2: EXERCISE & HEALTHY DIET

"We do communicate regularly about doing exercises, but to be acceptable for public, it needs to be practical."

System Stakeholder perspectives & responses

- *Regular circulation of IEC materials among the public on diet and exercise*
- *Awareness creation on methods like the diabetic plate model*
- *Training for health workers on right techniques of exercises*

- *Complexity of technical information in available materials*
- *Motivating beneficiaries towards regular exercise, beyond household chores*
- *Lack of aids to facilitate easy communication*

GENERIC CLINICAL PERSPECTIVES

- Healthy diet and exercise together effectively control hypertension and diabetes.
- Calorie-controlled, nutritious, balanced diets are essential for control of hypertension, diabetes, and general health.
- Exercise strengthens the heart and boosts circulation; healthy diet cuts excess fat—together, they improve blood pressure and heart function.
- Physical activity enhances insulin action; balanced meals prevent excess starch/sugar storage as fat.
- Muscles consume glucose during exercise, lowering blood sugar
- Fiber-rich, portion-controlled diets avoid glucose spikes.
- Excess starch-rich diets cause fat buildup around vital organs; exercise burns this stored fat, and curbs central obesity.
- Healthy eating limits arterial fat deposits
- Exercise improves blood flow, cutting risks of blockages, heart attacks, and strokes.
- Exercise reduces arterial stiffness
- Low calorie, low-fat, low-salt diets enable long-term blood pressure control.

Embodied knowledge

Facts

- Daily household chores, walking for tea or snacks, and light physical activity are sufficient exercises
- Walking or small movements during the day help maintain health and control blood pressure and sugar
- Combining daily activity with mindful eating is believed to support heart health, prevent weight gain, and improve overall well-being
- Even after following strict diet, BP or blood sugar, remains uncontrolled among few, so diet changes are not effective

NATURALLY ATTAINED

Myths

- Drinking natural remedies like fenugreek decoction, pappaya juice, or bitter gourd juice can control diabetes
- Eating rice and cereals as staple food does not cause any issue; there is no need to reduce rice/cereal intake
- Switching from rice to chapathi will control diabetes, and the quantity of chapathi does not matter
- Fruits are not good for people with diabetes
- Eggs are harmful for those with hypertension
- Lean individuals will not get diabetes and can eat anything without risk

EXAMPLE 2: EXERCISE & HEALTHY DIET

Extracts from Transformed Knowledge

Knowledge

Reduce starch intake gradually

- Starchy foods/ carbohydrates are the major source of energy for our body. Excess starch beyond our bodily energy requirements gets converted into fat. This fat gets deposited around the organs in the gut. It leads to weight gain and an increase in waist size. It affects the functions of these organs. It affects the body's proper use of insulin

Consumption of fruits and vegetables are crucial

- They are rich in fiber, vitamins, and minerals.
- Fiber slows sugar absorption; this helps in controlling blood sugar. It also improves insulin action.
- Low in fat and calories, makes them ideal for diabetes and hypertension control.
- People with diabetes need to take fruits in moderation, fruits with lower sugar content (glycemic index) (eg:: guava, pears, plums, apples, oranges) or locally available fruits in moderation.
- This helps prevent sudden spikes/ increase in blood sugar levels

Excess salt raises blood pressure and increases heart disease risk

- Deep-fried items, bakery products, and oily snacks raise cholesterol levels
- High-fat foods increase risk of heart disease and contribute to weight gain

Consistency is key: diet changes should be maintained for lasting benefits

Action

Reduce starch intake gradually

- Reduce the amount of rice and wheat consumed; gradually and progressively over a period of time
- Increase consumption of fruits and vegetables
- Completely avoid use of direct sugar
- Stop using sugar in tea, coffee, juices, and other drinks
- When you have cravings for sugary foods, try to replace them with fruits or other healthy options

Reduce excessive salt intake

- Avoid foods with high salt content, such as dried fish, pickles, and salted snacks
- Avoid using extra salt beyond what is added during cooking
- Keep salt containers away from the table and dining areas

Develop disciplined eating habits

- Eat meals at regular intervals to maintain stable energy and blood sugar
- Limit high-fat and fried foods
- Avoid buying and storing snacks at home
- Keep unhealthy foods out of sight
- Avoid late-night snacking
- Avoid snacking while watching TV or using your mobile phone
- Drink adequate water throughout the day
- Reduce visits to tea shops, bakeries and hotels where oily snacks and sugary drinks are common

Choose healthier alternatives

- Replace unhealthy snacks with fruits, nuts, or pulses.
- Read labels and choose low-fat, low-sugar, and low-salt items in stores

Build long-term healthy eating behavior

- Encourage family members, especially children, to follow healthy eating patterns
- Gradually shift from unhealthy habits to balanced meal plans

EXAMPLE 2: EXERCISE & HEALTHY DIET

Extracts from Transformed Knowledge

Knowledge

Benefits from exercise depend on how hard you work (intensity), how often you do it (frequency), and how long you do it (duration)

- The positive effects of exercise increase when you are consistent or regular
- Exercise would not be effective if you are into occasional activity

Exercise improves insulin function

- Physical activity helps the body use insulin more effectively, which helps control diabetes better
- When you exercise, muscles use glucose for energy, lowering blood sugar levels naturally
- Regular activity improves insulin production, absorption, and response, which means proper usage of insulin, an important benefit for diabetes control

Supports the health of heart

- Exercise strengthens the heart muscles, improves blood circulation, and reduces blood pressure
- Helps maintain normal blood pressure and prevents heart disease

Helps in weight and fat reduction

- Physical activity burns fat stored around internal organs
- Reduces waist circumference, which lowers the risk of diabetes and hypertension

Improves mental and overall well-being

- Exercise reduces anxiety and stress; improves sleep, increases energy levels, and enhances mood
- It strengthens bones, improves stamina and breathing capacity

Action

Aim for daily physical activity

- At least 30 minutes a day (150 minutes per week) significantly improves the health of your heart and reduces the risk of diabetes and hypertension
- Walking, cycling, slow jogging, or brisk walking are effective options

Step count matters

- Maintaining a daily step count of 8000-10,000 steps helps in lowering the risk of heart problems
- Even adding 100 extra steps contributes to better health
- Mobile phones or simple counting methods, or any digital material (smart watch) can be used to track steps

Ways to be more active

- Prefer using stairs whenever possible to using elevators
- Get off public transport/buses one stop earlier and walk
- Walk to nearby stores, public places, and places of worship
- Make use of parks, grounds, tracks, and community spaces
- Exercise in pairs or groups for better motivation
- Try walking rhythmically at a steady pace
- Warm up before doing exercise and cool down afterwards

Warming Up and Cooling Down: Why They Matter

- When you exercise, your body needs a gradual transition both at the start and the end

Warm-Up: Preparing Your Body

- A warm-up is the process of getting your body ready for the activity
- It is a few minutes of light activity performed before you start your actual workout
- It gradually increases your heart rate and blood circulation, delivering oxygen to your muscles
- It makes your muscles more flexible, reducing the risk of strains and injuries
- A Simple Example: If you plan to walk briskly for 30 minutes, you should spend the first 5 minutes walking at a slow, easy pace. This gets your muscles and heart ready for the faster pace
- For Beginners: If you are new to exercise, don't rush into intense workouts. Start with short, easy sessions (like walking a short distance) and be consistent

The Cool-Down: Easing Back to Normal

- A cool-down is the process of safely bringing your body back to its normal state after you finish exercising
- What it is: A few minutes of very light movement, often followed by stretching, performed immediately after your main workout
- Why it's important: It prevents you from stopping abruptly (which can make you feel dizzy or lightheaded)
- It helps your heart rate, breathing, and blood pressure return to their pre-exercise levels smoothly
- The Goal: To get your heart rate and pulse back to your normal resting rate, and for your breathing to become comfortable and regular again

EXAMPLE 3: SMOKING AND ALCOHOL CESSATION

"Smoking is considered so normal; only if they get to have a cough, they find it an issue, but never for hypertension"

System Stakeholder perspectives & responses

- *IEC materials are key for communication in NCD programs*
- *Ensuring availability and information on critical access points like de-addiction centres*

- *Lack of methods to teach smoking and alcohol cessation to beneficiaries*
- *Stigma among public is the major hurdle*

GENERIC CLINICAL PERSPECTIVES

- Tobacco and alcohol significantly increase the risk of developing diabetes, hypertension, and related complications.
- Nicotine elevates heart rate and raises blood pressure, worsening cardiovascular risk.
- Nicotine interferes with insulin action, increasing insulin resistance and contributing to poor glycemic control.
- Regular alcohol use raises blood sugar levels and increases the long-term risk of diabetes.
- In patients already diagnosed with diabetes or hypertension, tobacco and alcohol accelerate disease progression and increase the likelihood of severe outcomes.
- Repeated nicotine exposure leads to strong physical and psychological dependence, making quitting difficult without structured support.
- Counselling should emphasize that relapse during quitting attempts is common and not a failure—persistence and support are essential.

Embodied knowledge

Facts

- "I can quit anytime," not realising how quickly nicotine creates strong physical and mental dependence
- Quitting smoking and engaging in physical activity will reverse the affects of smoking
- Peer pressure and stress are acceptable reasons to smoke or drink
- Many avoid counselling, believing they already know enough or don't need help

NATURALLY ATTAINED

Myths

- One or two cigarettes are fine
- Smoking helps concentration
- Smoking augment digestion.
- Switching to lighter cigarettes, e-cigarettes will reduce the harmful effects
- Occasional drinking is safe
- Alcohol reduces stress
- Alcohol, when taken in small amounts, is good for the heart.
- Alcohol doesn't affect diabetes or BP if taken after food.

EXAMPLE 3: SMOKING AND ALCOHOL CESSATION

Extracts from Transformed Knowledge

Knowledge

- People who smoke or drink alcohol get diseases more easily, and those diseases can get worse. Smoking makes your heart beat faster and raises blood pressure, which messes up insulin and makes it harder to control blood sugar. Alcohol does something similar and makes these problems even bigger
- Nicotine is dangerous because it speeds up your heartbeat and raises blood pressure, which harms the heart. It also affects insulin, making blood sugar control difficult. Smoking delivers nicotine repeatedly, creating a strong urge to smoke again and again. This leads to physical and mental addiction, making it tough to quit. Stopping is key; better late than never
- Peer pressure and friends' influence can strongly affect your habits
- The urge or wish to smoke or drink usually lasts only a few minutes
- Understand that slipping from your decision to quit is not a failure at all

Action

- Making a firm decision to quit is the most critical first step—it's what sets everything in motion. You might fail a few times along the way, and that's completely normal; don't let it discourage you. Instead of trying to stop abruptly, which can be overwhelming, reduce your intake gradually and stop completely over time.
- Start by noting your current daily use, like how many cigarettes you smoke each day or how much alcohol you drink in ml or pegs.
- Pick a specific date as your target for quitting entirely.
- Set clear targets for each interval, such as reducing by a certain amount each week, and track how you're progressing by noting it down daily.
- Recording your consumption this way helps convince yourself of your commitment, keeps you motivated, shows your progress clearly, and lets you improvise or adjust as needed to reach your goal.
- It also helps you understand your triggers, like stress smoking or occasional binge drinking, so you can avoid or handle those situations better.
- If you slip in between and go back to your old habits, don't see it as a total failure, note down why you wanted to smoke or drink at that moment, understand the real reason behind it, and deal with that reason directly to stay on track.
- Do not keep cigarettes, tobacco, alcohol, ashtrays, or lighters at home to remove temptation. Ask your family members to help you clear out these items completely.
- avoid friends or situations that encourage smoking or drinking.
- As the urge or wish to smoke or drink lasts only for few minutes; hang in there, and it will pass.
- Distract yourself right away with simple activities like going for a walk, doing some physical exercise, or squeezing a stress ball.
- Use healthy substitutes like chewing cardamom or gooseberry whenever a craving hits to ease the urge.
- pick up your quitting plan and continue forward without losing heart.
- Lean on your family and healthcare providers for encouragement and guidance whenever you need it. If things feel tough, visit nearby de-addiction centers for specialized support. Use nicotine gum, inhalers, or tablets only after getting advice from a doctor to make sure it's safe for you.

References

1. Department of Health and Family Welfare, Govt of Kerala. (2023) Government order on redefining the activities of Health and Wellness Centers and roles and roles and responsibilities of stakeholders *(G.O.No. 131/2023/H&FWD)* [Government Order in Malayalam]
2. Kerala State Health Systems Resource Centre. (2025). *Guidelines for expanded healthcare services at Janakeeya Arogya Kendrams*. State Health Systems Resource Centre, Kerala.
3. Graham ID, Logan J, Harrison MB, Straus SE, Tetroe J, Caswell W, et al. Lost in knowledge translation: time for a map? *J Contin Educ Health Prof*, 2006;26(1):13-24.
4. Greenhalgh T, Robert G, Macfarlane F, Bate P, Kyriakidou O. Diffusion of innovations in service organizations: systematic review and recommendations. *Milbank Q*, 2004;82(4):581-629.
5. Gibbons M, Limoges C, Nowotny H, Schwartzman S, Scott P, Trow M. The New Production of Knowledge: The Dynamics of Science and Research in Contemporary Societies. London: Sage Publications; 1994.
6. Muntaner, C., Chung, H., Murphy, K., & Ng, E. (2012). Barriers to knowledge production, knowledge translation, and urban health policy change: Ideological, economic, and political considerations. *Journal of Urban Health, 89*(6), 915–924.
7. Jeemon, P., Harikrishnan, S., Ganapathi, S., Sivasankaran, S., Binukumar, B., Padmanabhan, S., Tandon, N., & Prabhakaran, D. (2021). Efficacy of a family-based cardiovascular risk reduction intervention in individuals with a family history of premature coronary heart disease in India (PROLIFIC): an open-label, single-centre, cluster randomised controlled trial. *The Lancet. Global health*, *9*(10), e1442–e1450.
8. Thankappan, K. R., Sathish, T., Tapp, R. J., Shaw, J. E., Lotfaliany, M., Wolfe, R., Absetz, P., Mathews, E., Aziz, Z., Williams, E. D., Fisher, E. B., Zimmet, P. Z., Mahal, A., Balachandran, S., D'Esposito, F., Sajeev, P., Thomas, E., & Oldenburg, B. (2018). A peer-support lifestyle intervention for preventing type 2 diabetes in India: A cluster-randomized controlled trial of the Kerala Diabetes Prevention Program. *PLoS medicine*, *15*(6), e1002575.
9. Ritchie, J., & Lewis, J. (Eds.). (2003). Qualitative research practice: A guide for social science students and researchers. Sage Publications.
10. Parasuraman, G., Jeemon, P., Thankappan, K. R., Ali, M. K., Mahal, A., McPake, B., Chambers, J., Absetz, P., Thirunavukkarasu, S., Nabil, A. M., Kripa, S. V. S., Akshay, P. K., Ayyasamy, L., Nambirajan, M. K., Ramalingam, A., Nagarajan, R., Shrestha, A., Gopal, B., Selvam, J. M., Haregu, T., & Oldenburg, B. (2024). Community Control of Hypertension and Diabetes (CoCo-HD) program in the Indian states of Kerala and Tamil Nadu: A study protocol for a type 3 hybrid trial. *BMC Public Health, 24*, Article 2275
11. Morrow, S. L. (2005). Quality and trustworthiness in qualitative research in counseling psychology. *Journal of Counseling Psychology, 52*(2), 250–260.
12. Spall, S. (1998). Peer debriefing in qualitative research: Emerging operational models. *Qualitative Inquiry, 4*(2), 280–292.
13. Morrow, S. L. (2005). Quality and trustworthiness in qualitative research in counseling psychology. *Journal of Counseling Psychology, 52*(2), 250–260.
14. DiMaggio PJ, Powell WW. The iron cage revisited: institutional isomorphism and collective rationality in organizational fields. *Am Sociol Rev*, 1983;48(2):147-60.
15. May C, Finch T. Implementing, embedding, and integrating practices: an outline of Normalization Process Theory. *Sociology*, 2009;43(3):535-54.
